# Association between whole grain intake and all-cause mortality: a meta-analysis of cohort studies

**DOI:** 10.18632/oncotarget.11491

**Published:** 2016-08-22

**Authors:** Xiao Ma, Wei-Guo Tang, Yang Yang, Qing-Li Zhang, Jia-Li Zheng, Yong-Bing Xiang

**Affiliations:** ^1^ State Key Laboratory of Oncogenes and Related Genes and Department of Epidemiology, Shanghai Cancer Institute, Renji Hospital, Shanghai Jiaotong University School of Medicine, Shanghai, China; ^2^ Department of Epidemiology and Biostatistics, Arnold School of Public Health, University of South Carolina, Columbia, SC 29208, USA

**Keywords:** diet, whole grain intake, mortality, meta-analysis, prospective cohort studies

## Abstract

Some observational studies have examined the association between dietary whole grain intake and all-cause mortality, but the results were inconclusive. We therefore conducted a meta-analysis to summarize the evidence from cohort studies regarding the association between whole grain intake and all-cause mortality. Pertinent studies were identified by searching PubMed, Embase and Web of Knowledge, up to February 28, 2016. Study-specific estimates were combined using random-effects models. Eleven prospective cohort studies involving 101,282 deaths and 843,749 participants were included in this meta-analysis. The pooled relative risk of all-cause mortality for the highest category of whole grain intake versus lowest category was 0.82 (95% confidence interval: 0.78, 0.87). There was a 7% reduction in risk associated with each 1 serving/day increase in whole grain intake (relative risk = 0.93; 95% confidence interval: 0.89, 0.97). No publication bias was found. This analysis indicates that higher intake of whole grain is associated with a reduced risk of all-cause mortality. The findings support current recommendations for increasing whole grain consumption to promote health and overall longevity.

## INTRODUCTION

The number of deaths was 52.8 million globally in 2010 and the deaths from non-communicable diseases rose from just under 8 million in 1990 to 34.5 million in 2010, accounting for two of every three deaths [1]. Of chronic non-communicable diseases, cardiovascular disease, and cancer are among the main causes of death [1]. A high quality diet including plant foods is one of the most promising factors in primary and secondary prevention of non-communicable diseases [2].

Grains, also called cereals, are the seeds of grasses cultivated for food. Whole grains are the entire seed of a plant and this seed (which industry calls a “kernel”) is made up of three key edible parts- bran, germ, and endosperm [3]. The bran and germ, removed during the milling process, are rich in dietary fiber, protein, micronutrients and phytochemicals [4]. Whole grains have been widely recommended in dietary guidelines as healthful food [5, 6], and have shown consistently favorable effects on insulin sensitivity [7, 8], lipid profile [9], endothelial function [10], antioxidant activity [11] and inflammation [12, 13]. The Dietary Guidelines for Americans therefore recommend at least an intake of 3 servings of whole-grain products per day [6], but the mean intake of whole grains in the United States is less than 1 serving/d [14].

Accumulating epidemiological evidence indicates that high intake of whole grains might decrease the risks of obesity/abdominal fatness [15], type 2 diabetes [16], hypertension [17], cardiovascular disease [16] and major cancers [18–22]. Extensive prospective cohort studies also evaluated the association between dietary whole grain intake and all-cause mortality in general population [23–32] and specific disease-related population [33, 34]. The evidence from prospective cohort studies on dietary whole grain intake in relation to all-cause mortality has not yet been summarized, we therefore conducted a meta-analysis of prospective cohort studies to quantify this association.

## RESULTS

### Literature search

**Figure 1:**
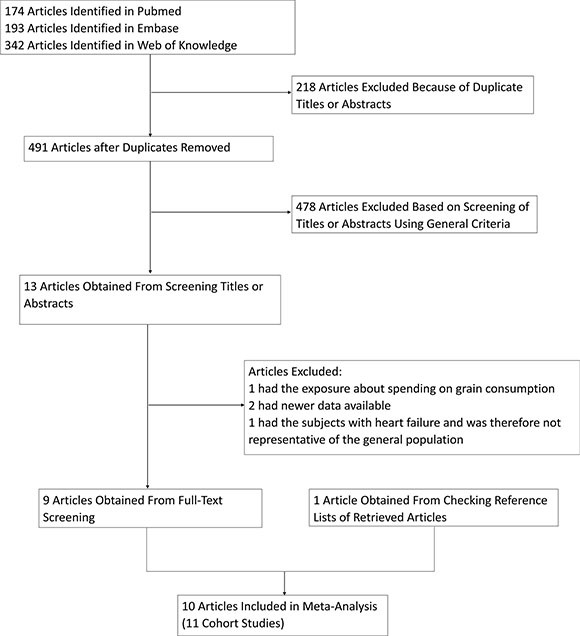
Process used to select prospective cohort studies for a meta-analysis of the association between whole grain intake and all-cause mortality, 1964–2016

A flow chart for the search process is presented in Figure 1. Our search strategy identified 709 potentially relevant articles from the 3 databases, and 218 records were excluded because they were duplicates. After a review of the titles and abstracts based on the pre-specified inclusion and exclusion criteria, 478 articles were further excluded. After reviewing the full text of the remaining thirteen articles, four studies were excluded because 1) the exposure of interest was the spending on grain consumption expenses (*n* = 1) [35]; 2) newer data was available (*n* = 2) [34, 36]; and 3) subjects were heart failure patients and therefore not representative of the general population (*n* = 1) [33]. One study [27] that was identified by checking the reference lists of retrieved articles was also included, giving a total of ten articles with eleven independent prospective cohort studies in the final analysis [23–32].

### Study characteristics

Eleven prospective cohort studies from ten publications [23–32] published between 2001 and 2016, including 843,749 individuals and 101,282 deaths, were eligible for the present meta-analysis (Supplementary Table S1). Two Harvard cohorts were included in one publication [32]. Ten cohort studies used food frequency questionnaire (FFQ) to assess dietary whole grains [23, 24, 26–32] and the remaining one used 3-day food record [25]. The included studies were conducted in the United States (*n* = 7) [24, 26, 27, 29, 30, 32] and Europe (*n* = 4) [23, 25, 28, 31]. The sample sizes ranged from 535 [25] to 367,442 [30], and the median follow-up time varied from 5.9 years [28] to 26 years [32]. All of the included studies adjusted for age, smoking status, total energy intake, and most of them included adjustment for other potential confounders, such as gender (if available), body mass index (BMI), physical activity level and alcohol drinking, etc.

### Whole grain intake and all-cause mortality

#### Categorical meta-analysis

**Figure 2:**
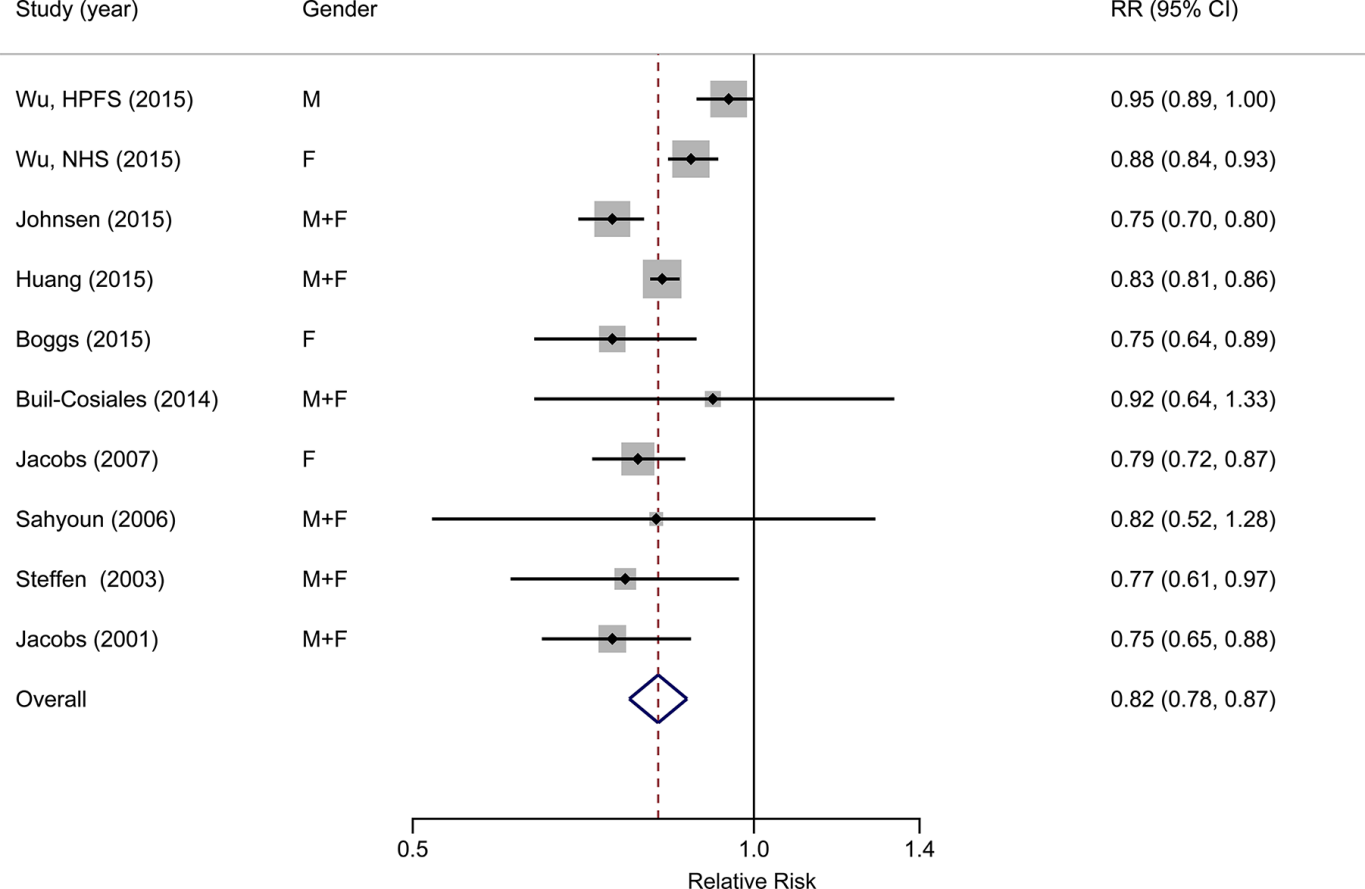
Forest plot (random-effects model) of the association between whole grain intake (highest category vs. lowest) and all-cause mortality Black points indicate study-specific RRs (the size of the square reflects the study-specific statistical weight); horizontal lines indicate 95% CIs; the diamond indicates the summary estimate with its 95% CI.

Ten studies from nine publications [23–26, 28–32] with a total of 722,897 individuals and 91,591 deaths were included in the categorical meta-analysis. In combined estimates for the highest versus the lowest category, whole grain intake was inversely associated with all-cause mortality risk (RR = 0.82, 95% CI: 0.78, 0.87), with substantial heterogeneity among studies (*I*^2^ = 76.6%, *P*_heterogeneity_ < 0.001) (Figure 2). No significant publication bias was observed according to the funnel plot (Supplementary Figure S1), Begg's test (*P* = 0.858), Egger's test (*P* = 0.575).

#### Dose-response meta-analysis

**Figure 3:**
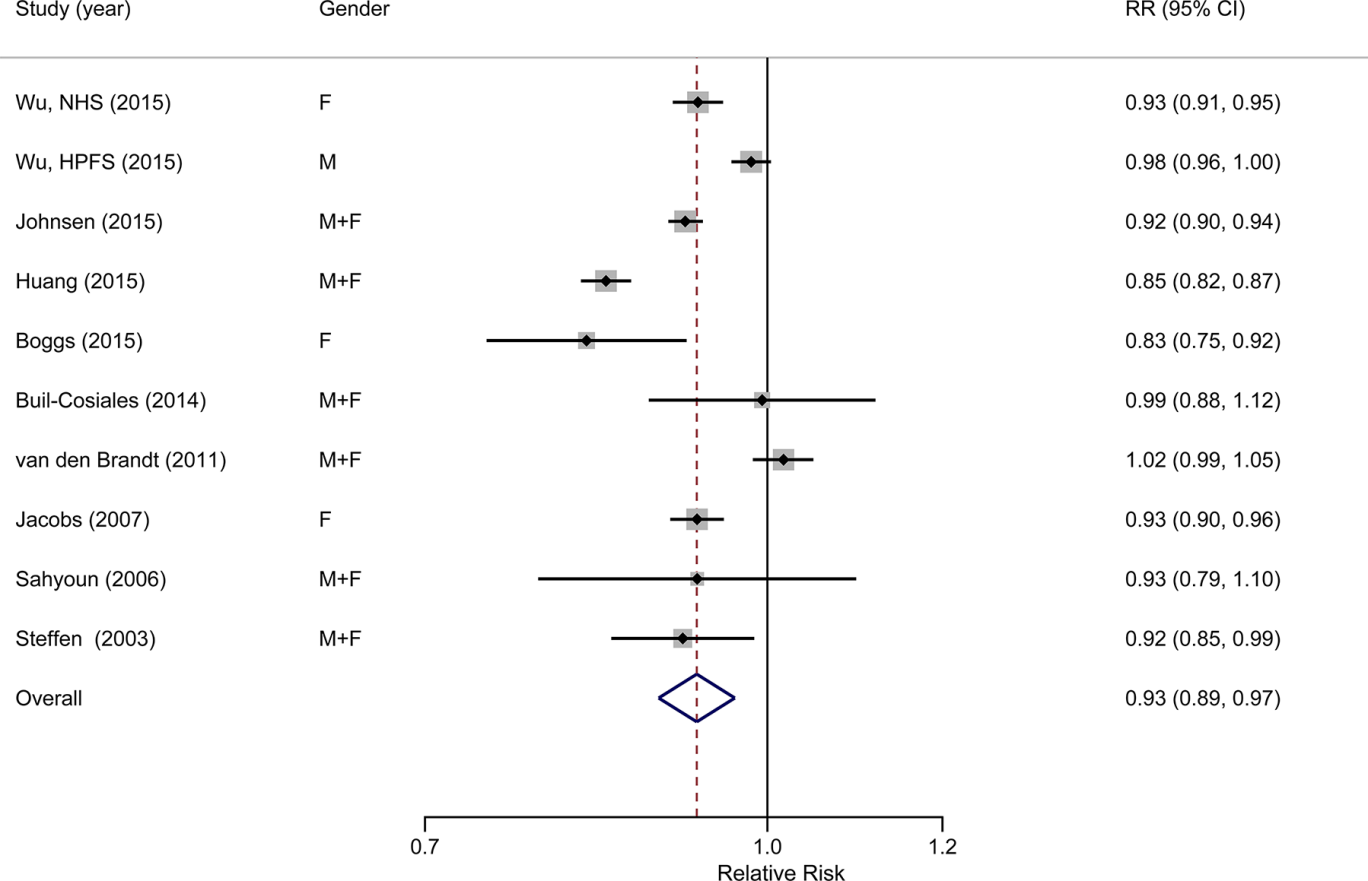
Forest plot (random-effects model) of the association between whole grain intake (each 1 serving/d increase) and all-cause mortality Black points indicate study-specific RRs (the size of the square reflects the study-specific statistical weight); horizontal lines indicate 95% CIs; the diamond indicates the summary estimate with its 95% CI.

Ten studies from nine publications [24–32] with a total of 809,901 individuals and 99,224 deaths were included in the dose-response meta-analysis. We used serving/d (serving per day) as the units. The pooled relative risk for a 1 serving/d increment of whole grain intake was 0.93 (95% CI: 0.89, 0.97), with significant heterogeneity (*I*^2^ = 92.4%, *P*_heterogeneity_ < 0.001) (Figure 3). No significant publication bias was observed according to the funnel plot (Supplementary Figure S2), Begg's test (*P* = 0.858), Egger's test (*P* = 0.895).

### Subgroup and sensitivity analyses

In subgroup analyses of the association between whole grain intake and all-cause mortality, all estimates, despite disparate magnitudes, showed inverse associations, and all were statistically significant except in male population (Table 1). Moreover, the inverse association was much stronger when the analysis was restricted to the population in Europe (RR = 0.75; 95% CI: 0.71, 0.80; *I*^2^ = 0%, *P*_heterogeneity_ = 0.558) compared with the pooled relative risk for the association of dietary whole grain intake with all-cause mortality among population in America. For studies in which with adjustment for BMI, physical activity level, alcohol consumption, diabetes, blood pressure, or serum cholesterol level, the pooled relative risk was higher than that in studies without adjustment. However, meta-regression analysis showed that no variables may account for the heterogeneity across studies on the association between whole grain intake and total mortality.

**Table 1:** Pooled relative risk of all-cause mortality for persons in the highest category of whole grain intake versus those in the lowest category in a meta-analysis, by study characteristic, 2001–2016

	Studies	Summary RR	95% CI	*I*^2^, %	*P* for heterogeneity*	*P* for heterogeneity**
	*n*					
All studies	10	0.82	(0.78, 0.87)	76.6	< 0.001	
Gender						0.725
Male	2	0.85	(0.67, 1.07)	94.3	< 0.001	
Female	4	0.80	(0.72, 0.88)	77.3	0.004	
Male + Female	5	0.83	(0.80, 0.85)	0	0.673	
Study location						0.143
America	7	0.85	(0.80, 0.90)	74.4	< 0.001	
Europe	3	0.75	(0.71, 0.80)	0	0.558	
Follow-up time^†^						0.686
> 15	5	0.83	(0.75, 0.91)	88.1	< 0.001	
≤ 15	5	0.82	(0.80, 0.85)	0	0.673	
Cohort size^‡^						0.388
> 35,000	5	0.84	(0.78, 0.90)	88.1	< 0.001	
≤ 35,000	5	0.78	(0.73, 0.84)	0	0.886	
Adjustment for:						
BMI						0.707
Yes	8	0.83	(0.76, 0.90)	80.0	< 0.001	
No	2	0.82	(0.76, 0.88)	28.8	0.236	
Physical activity level						0.241
Yes	9	0.84	(0.79, 0.89)	69.5	0.001	
No	1	0.75	(0.70, 0.80)	NA	NA	
Alcohol consumption						0.411
Yes	9	0.83	(0.78, 0.88)	77.9	< 0.001	
No	1	0.75	(0.65, 0.88)	NA	NA	
Diabetes^§^						0.253
Yes	8	0.84	(0.79, 0.89)	73.3	< 0.001	
No	2	0.75	(0.70, 0.80)	0	0.701	
Blood pressure						0.090
Yes	5	0.87	(0.80, 0.94)	64.6	0.023	
No	5	0.79	(0.75, 0.84)	54.1	0.069	
Serum cholesterol level						0.063
Yes	3	0.88	(0.80, 0.96)	79.2	0.008	
No	7	0.79	(0.76, 0.83)	35.9	0.154	

BMI, body mass index; CI, confidence interval; NA, not available; RR, relative risk.

* *P* value for heterogeneity within each subgroup.

** *P* value for heterogeneity between subgroups in meta-regression analysis.

† The median follow-up time for studies included in the meta-analysis was 15 years.

‡ The median cohort size for studies included in the meta-analysis was 35,000 participants.

§ Studies excluding prevalent diabetes at baseline were included in the “yes” group.

In sensitivity analyses, we sequentially excluded one study at a time and reanalyzed the remaining data. The pooled relative risks for sensitivity analyses were still statistically significant and similar to the overall estimate. The pooled relative risks for all-cause mortality ranged from 0.81 (95% CI: 0.77, 0.85) when the Health Professionals Follow-up Study by Wu et al. [32] was excluded, to 0.84 (95% CI: 0.79, 0.89) when the Norwegian Women and Cancer Study, Northern Sweden Health and Disease Study, Danish Diet, Cancer and Health Study by Johnsen et al. [31] was excluded.

## DISCUSSION

To our knowledge, the present study is the first meta-analysis quantitatively assessing the association between dietary whole grain intake and all-cause mortality. In this meta-analysis, increased whole grain intake was significantly associated with a reduced risk of total death. The risk of all-cause mortality was decreased by 18% for individuals in the highest category intake of whole grain, compared with it in the lowest. The results of the dose-response meta-analysis suggested that each additional 1 serving whole grain intake daily may lower the risk of all-cause mortality by 7%.

The protective effect of whole grain consumption on risk of all-cause mortality is biologically plausible. Whole grains are rich source of fiber, vitamins, minerals, phenolic compounds, phytoestrogens, and other phytochemicals, which may potentially explain whole grains' favorable effects [11, 37–42]. Many of these compounds are redox-active secondary plant metabolites [39, 40, 43, 44] that are produced by plants to protect against oxidative and other types of stress. These components can activate defense-related genes in the cells of plants to support the antioxidant defense and thereby reduce the damaging effects of chronic inflammation via several mechanisms [40, 45]. It has been suggested that these compounds can also mount an antioxidant defense in human body cells by inducing expression of similar genes for antioxidant and detoxification enzymes [40, 46, 47]. Therefore, a number of previous meta-analyses observed that whole grain intake was associated with decreased risk of major chronic diseases, including cardiovascular disease (CVD) [48–50], diabetes [51–53], and common cancers [18, 19, 21, 54]. Our findings are concordant with these meta-analyses of whole grain intake in relation to the risk of chronic diseases.

In subgroup analyses, among male population, no significant inverse association was found. This might be due to that the number of cohorts in the group was only two, so the limited sample sizes had inadequate test power to examine the association. Most studies included in the present meta-analysis adjusted for some of the major potential confounders or risk factors, such as BMI, physical activity level, alcohol consumption. When we stratified studies by adjustment for specific confounders, the magnitudes of the association in the subgroups with adjustment were considered to be more reliable because these factors may be confounders in the analysis due to their associations with both the risk of all-cause mortality and whole grain consumption. When we restricted the analysis to studies that were adjusted for diabetes, blood pressure, or serum cholesterol level, the pooled relative risk was more close to 1 than the overall estimate. Dietary whole grain intake could influence the risk of all-cause mortality via several different mechanisms. Controlling for any of the intermediate variables, such as diabetes, blood pressure, or serum cholesterol level, in the hypothesized casual pathway between dietary whole grain and all-cause mortality might lead to overadjustment and thus bias the result towards null [55]. Therefore, the true association between dietary whole grain intake and all-cause mortality may be even stronger.

Although most studies indicated inverse association between whole grain consumption and all-cause mortality, significant heterogeneity was observed in this meta-analysis, which could limit the interpretability of the pooled estimate. There were several potential explanations for the observed between-study heterogeneity. First, the way in which these articles compared categories was different. Several studies calculated the estimation for the highest quintile vs. the lowest [23, 24, 26, 29, 30, 32], while others calculated for the highest quartile vs. the lowest [25, 31], and another [28] used the highest quintile vs. the lowest two quintiles. Second, the cut-off points for each category were inconsistent, which could certainly have contributed to inter-study differences in the strength of the observed associations, especially in the Europe studies by Buil-Cosiales et al. [28] and Johnsen et al. [31]. This is due to that Europeans are recommended to have more whole grain intake than Americans. In the Scandinavian countries at least 75 grams per day of whole grain intake which equals approximately 131 grams per day (4.7 servings/day) of whole grain products is recommended [56] while in the USA and Canada the recommendation is that “all adults eat at least half their grains as whole grains” so at least 48 grams whole grains or 84 grams whole grain products should be consumed per day (3 servings/day) [6]. Third, we cannot rule out the possibility that the study locations, the various study follow-up periods, the different sample sizes of the cohorts, or the potential confounders, intermediate variables for which the researchers adjusted might also have contributed to some of the heterogeneity reported herein.

The present meta-analysis had some strengths. To the best of our knowledge, this was the first meta-analysis to explore the relationship between dietary whole grain intake and all-cause mortality. A highlight of this present meta-analysis was the prospective design of the included cohort studies, which should have greatly reduced the potential selection and recall bias. The large study population (101,282 deaths among 843,749 participants) enabled us to provide sufficient statistical power to quantitatively assess the relationship between whole grain intake and all-cause mortality. Most of the studies included in the meta-analysis adjusted for large number of major confounders. Because of this, the pooled estimate may be less susceptible to confounding bias.

However, some limitations of the meta-analysis should be considered. First, it was difficult to consistently define and precisely evaluate the intake of whole grains or whole grain products in epidemiological studies and some degree of measurement error was inevitable, and reporting whole grain intakes as the actual amount of whole grain intake per dry weight had been pointed out [57]. Most studies reported whole grain food intake as the amount of whole grain products (fresh weight), while only two articles reported intakes in actual amount of whole grain (dry weight) [31, 32]. Moreover, the study by Johnsen et al. reported results for both whole grain products and actual whole grain intake [31]. Some studies and guidelines have classified some products (e.g., breakfast cereals) as whole grain foods if they have a whole grain content of ≥ 25% [24, 26, 30] or ≥ 51% [58] of the weight of the product, leading to misclassification of the exposure. In cohort studies, non-differential misclassification of exposure leads to an underestimation of the magnitude of the associations. In this respect, the observed reductions in mortality associated with whole grain intake may be conservative estimates. Second, small-sample bias, such as publication bias, might have influenced the results. Although there was no evidence of publication bias in the present meta-analysis, tests for publication bias had low statistical power, especially when the number of studies was limited. Third, because the assessment was based on observational studies, we could not rule out the possibility that unknown residual confounding might still have affected the results in each study and thus the pooled estimates of the meta-analyses. Last but not least, the studies in the present meta-analysis were all conducted in Europe and America with no study conducted in Asia. This might somewhat limit the generalizability of the results from this meta-analysis.

In conclusion, the present meta-analysis indicates that an increased whole grain intake is associated with a reduced risk of all-cause mortality. These findings add to and extend the evidence that an increased dietary whole grain intake may exert healthy effects and decrease the risk of all-cause mortality and support current recommendations to increase whole grain consumption to promote health and overall longevity.

## MATERIALS AND METHODS

### Literature search

We followed standard criteria for reporting meta-analyses of observational studies [59]. We performed a systematic literature search and review of peer-reviewed articles published on PubMed, Embase, and Web of Knowledge through February 2016 using the following key words: (grain OR grains) AND (mortality OR death) AND (prospective OR longitudinal OR cohort OR cohorts OR follow-up). The identified publications were reviewed independently for their relevance to the research topic by two authors (X.M., W.-G.T.). We also searched the reference lists of relevant publications to include eligible studies.

### Study selection

Published studies were included if they 1) reported all-cause/total mortality as the outcome of interest; 2) were conducted in a general population; 3) used prospective cohort design; 4) presented information on whole grain intake as the exposure of interest; and 5) provided estimates of relative risk (RR) or hazard ratio (HR) with confidence intervals (CIs) or standard errors or the data necessary to calculate these estimates.

The studies were excluded if they 1) were non-English language; 2) were not original articles, such as reviews, letters, comments, etc.; and 3) had repetitive data on the same population. We used the most recent report or report with the largest number of cases if there were duplicates.

### Data extraction

Data abstracted from each study were as follows: first author's name, year of publication, country in which the study was conducted, duration of follow-up, gender of the study population, age group of the subjects, sample size of the cohort, number of deaths, assessment tool used to measure dietary whole grains, type of exposure, categories of dietary whole grains and relative risks and 95% confidence intervals for all-cause mortality associated with those categories, and covariates included for adjustment in multivariable models as well.

### Statistical analysis

We calculated the pooled relative risk of all-cause mortality and its 95% confidence interval for the highest category of dietary whole grain intake versus the lowest using DerSimonian and Laird random-effects model, which incorporate both within- and between-study variations [60].

For the dose-response meta-analysis, numbers of deaths and persons/person-years for at least 3 whole grain intake categories and the mean or median values of the categories were needed. If the mean or median values were not reported in the studies, the estimated midpoints of the categories were used for substitution. When the highest categories were open-ended, we assumed that the open-ended categories were of the same amplitude as the adjacent categories [61].We used the method proposed by Greenland and Longnecker [62] and Orsini et al. [63] to compute the linear trend of correlated log RRs across categories of the dietary whole grain intake. Dose-response results in forest plots were presented on the basis of 1 serving per day increment for the dietary whole grain intake. According to the recommendations by The Whole Grains Council [64] and Ross et al. [57], one serving dietary whole grain intake is either one ounce (28-g) of a 100% whole grain food in its ready-to-eat form or the amount of food containing 16-g of whole grain ingredients (28 g of whole-grain products approximates 16 g of whole grain). We converted the amount of whole-grain products and whole grain using gram as the unit into serving as follows: For studies [31, 32] reporting whole grains in grams, the intake was converted in servings, using 16 g as a serving size. For studies [27, 28] in which whole-grain products were reported in grams, intake was converted in servings, using 28 g as a serving size.

To assess the source of variations among studies, we carried out subgroup analyses stratified by gender, study location, follow-up period, and cohort size. We also conducted analyses stratified by whether studies adjusted for potential important confounders, including total energy intake, BMI, smoking status, alcohol consumption, physical activity level, or adjusted for potential intermediate variables, including diabetes, blood pressure, and serum cholesterol level. For each stratification variable, heterogeneity between subgroups was evaluated by conducting meta-regression analyses.

We conducted sensitivity analyses to assess the influence of individual studies by excluding one study at a time and created a sensitivity plot. Publication bias was assessed using Begg's test [65] and Egger's test [66]. Heterogeneity among studies was assessed with the *Q* and *I*^2^ statistics, and results were defined as heterogeneous for a *P* value < 0.10 or *I*^2^ > 50% [67].

All statistical analyses were conducted using Stata/SE software, version 12.0 (Stata-Corp LP, College Station, Texas). Two-sided *P* value < 0.05 was considered statistically significant unless otherwise specified.

## SUPPLEMENTARY MATERIALS FIGURES AND TABLES





## References

[1] Lozano R, Naghavi M, Foreman K, Lim S, Shibuya K, Aboyans V, Abraham J, Adair T, Aggarwal R, Ahn SY, Alvarado M, Anderson HR, Anderson LM (2012). Global and regional mortality from 235 causes of death for 20 age groups in 1990 and 2010: a systematic analysis for the Global Burden of Disease Study 2010. Lancet.

[2] Wagner KH, Brath H (2012). A global view on the development of non communicable diseases. Prev Med.

[3] The Whole Grains Council What is a Whole Grain?.

[4] Slavin J (2004). Whole grains and human health. Nutr Res Rev.

[5] European Food Information Council (EUFIC) Whole grain fact sheet.

[6] The Department of Agriculture (USDA) and the Department of Health and Human Services (HHS) (2005). Dietary Guidelines for Americans.

[7] McCarty MF (2005). Magnesium may mediate the favorable impact of whole grains on insulin sensitivity by acting as a mild calcium antagonist. Med Hypotheses.

[8] Pereira MA, Jacobs DR, Pins JJ, Raatz SK, Gross MD, Slavin JL, Seaquist ER (2002). Effect of whole grains on insulin sensitivity in overweight hyperinsulinemic adults. Am J Clin Nutr.

[9] Harland JI, Garton LE (2008). Whole-grain intake as a marker of healthy body weight and adiposity. Public Health Nutr.

[10] Katz DL, Nawaz H, Boukhalil J, Chan W, Ahmadi R, Giannamore V, Sarrel PM (2001). Effects of oat and wheat cereals on endothelial responses. Prev Med.

[11] Adom KK, Liu RH (2002). Antioxidant activity of grains. J Agric Food Chem.

[12] Qi L, Hu FB (2007). Dietary glycemic load, whole grains, and systemic inflammation in diabetes: the epidemiological evidence. Curr Opin Lipidol.

[13] Qi L, van Dam RM, Liu S, Franz M, Mantzoros C, Hu FB (2006). Whole-grain, bran, and cereal fiber intakes and markers of systemic inflammation in diabetic women. Diabetes Care.

[14] McKeown NM, Meigs JB, Liu S, Saltzman E, Wilson PW, Jacques PF (2004). Carbohydrate nutrition, insulin resistance, and the prevalence of the metabolic syndrome in the Framingham Offspring Cohort. Diabetes Care.

[15] Karl JP, Saltzman E (2012). The role of whole grains in body weight regulation. Adv Nutr.

[16] Ye EQ, Chacko SA, Chou EL, Kugizaki M, Liu S (2012). Greater whole-grain intake is associated with lower risk of type 2 diabetes, cardiovascular disease, and weight gain. J Nutr.

[17] Lillioja S, Neal AL, Tapsell L, Jacobs DR (2013). Whole grains, type 2 diabetes, coronary heart disease, and hypertension: links to the aleurone preferred over indigestible fiber. Biofactors.

[18] Aune D, Chan DS, Lau R, Vieira R, Greenwood DC, Kampman E, Norat T (2011). Dietary fibre, whole grains, and risk of colorectal cancer: systematic review and dose-response meta-analysis of prospective studies. BMJ.

[19] Lei Q, Zheng H, Bi J, Wang X, Jiang T, Gao X, Tian F, Xu M, Wu C, Zhang L, Li N, Li J (2016). Whole Grain Intake Reduces Pancreatic Cancer Risk: A Meta-Analysis of Observational Studies. Medicine (Baltimore).

[20] Mourouti N, Kontogianni MD, Papavagelis C, Psaltopoulou T, Kapetanstrataki MG, Plytzanopoulou P, Vassilakou T, Malamos N, Linos A, Panagiotakos DB (2016). Whole Grain Consumption and Breast Cancer: A Case-Control Study in Women. J Am Coll Nutr.

[21] Jacobs DR, Marquart L, Slavin J, Kushi LH (1998). Whole-grain intake and cancer: an expanded review and meta-analysis. Nutr Cancer.

[22] Kasum CM, Nicodemus K, Harnack LJ, Jacobs DR, Folsom AR, Iowa Women's Health S (2001). Whole grain intake and incident endometrial cancer: the Iowa Women's Health Study. Nutr Cancer.

[23] Jacobs DR, Meyer HE, Solvoll K (2001). Reduced mortality among whole grain bread eaters in men and women in the Norwegian County Study. Eur J Clin Nutr.

[24] Steffen LM, Jacobs DR, Stevens J, Shahar E, Carithers T, Folsom AR (2003). Associations of whole-grain, refined-grain, and fruit and vegetable consumption with risks of all-cause mortality and incident coronary artery disease and ischemic stroke: the Atherosclerosis Risk in Communities (ARIC) Study. Am J Clin Nutr.

[25] Sahyoun NR, Jacques PF, Zhang XL, Juan W, McKeown NM (2006). Whole-grain intake is inversely associated with the metabolic syndrome and mortality in older adults. Am J Clin Nutr.

[26] Jacobs DR, Andersen LF, Blomhoff R (2007). Whole-grain consumption is associated with a reduced risk of noncardiovascular, noncancer death attributed to inflammatory diseases in the Iowa Women's Health Study. Am J Clin Nutr.

[27] van den Brandt PA (2011). The impact of a Mediterranean diet and healthy lifestyle on premature mortality in men and women. Am J Clin Nutr.

[28] Buil-Cosiales P, Zazpe I, Toledo E, Corella D, Salas-Salvado J, Diez-Espino J, Ros E, Fernandez-Creuet Navajas J, Santos-Lozano JM, Aros F, Fiol M, Castaner O, Serra-Majem L (2014). Fiber intake and all-cause mortality in the Prevencion con Dieta Mediterranea (PREDIMED) study. Am J Clin Nutr.

[29] Boggs DA, Ban Y, Palmer JR, Rosenberg L (2015). Higher diet quality is inversely associated with mortality in African-American women. J Nutr.

[30] Huang T, Xu M, Lee A, Cho S, Qi L (2015). Consumption of whole grains and cereal fiber and total and cause-specific mortality: prospective analysis of 367,442 individuals. BMC Med.

[31] Johnsen NF, Frederiksen K, Christensen J, Skeie G, Lund E, Landberg R, Johansson I, Nilsson LM, Halkjaer J, Olsen A, Overvad K, Tjonneland A (2015). Whole-grain products and whole-grain types are associated with lower all-cause and cause-specific mortality in the Scandinavian HELGA cohort. Br J Nutr.

[32] Wu H, Flint AJ, Qi Q, van Dam RM, Sampson LA, Rimm EB, Holmes MD, Willett WC, Hu FB, Sun Q (2015). Association between dietary whole grain intake and risk of mortality: two large prospective studies in US men and women. JAMA Intern Med.

[33] Levitan EB, Lewis CE, Tinker LF, Eaton CB, Ahmed A, Manson JE, Snetselaar LG, Martin LW, Trevisan M, Howard BV, Shikany JM (2013). Mediterranean and DASH diet scores and mortality in women with heart failure: The Women's Health Initiative. Circ Heart Fail.

[34] He M, van Dam RM, Rimm E, Hu FB, Qi L (2010). Whole-grain, cereal fiber, bran, and germ intake and the risks of all-cause and cardiovascular disease-specific mortality among women with type 2 diabetes mellitus. Circulation.

[35] Lo YT, Chang YH, Wahlqvist ML, Huang HB, Lee MS (2012). Spending on vegetable and fruit consumption could reduce all-cause mortality among older adults. Nutr J.

[36] Jacobs DR, Meyer KA, Kushi LH, Folsom AR (1999). Is whole grain intake associated with reduced total and cause-specific death rates in older women? The Iowa Women's Health Study. Am J Public Health.

[37] Adom KK, Sorrells ME, Liu RH (2005). Phytochemicals and antioxidant activity of milled fractions of different wheat varieties. J Agric Food Chem.

[38] Miller HE, Rigelhof F, Marquart L, Prakash A, Kanter M (2000). Antioxidant content of whole grain breakfast cereals, fruits and vegetables. J Am Coll Nutr.

[39] Halvorsen BL, Holte K, Myhrstad MC, Barikmo I, Hvattum E, Remberg SF, Wold AB, Haffner K, Baugerod H, Andersen LF, Moskaug O, Jacobs DR, Blomhoff R (2002). A systematic screening of total antioxidants in dietary plants. J Nutr.

[40] Blomhoff R (2005). Dietary antioxidants and cardiovascular disease. Curr Opin Lipidol.

[41] Yang Y, Zhao L-G, Wu Q-J, Ma X, Xiang Y-B (2015). Association Between Dietary Fiber and Lower Risk of All-Cause Mortality: A Meta-Analysis of Cohort Studies. American Journal of Epidemiology.

[42] Kim Y, Je Y (2014). Dietary fiber intake and total mortality: a meta-analysis of prospective cohort studies. Am J Epidemiol.

[43] Dragland S, Senoo H, Wake K, Holte K, Blomhoff R (2003). Several culinary and medicinal herbs are important sources of dietary antioxidants. J Nutr.

[44] Halvorsen BL, Carlsen MH, Phillips KM, Bohn SK, Holte K, Jacobs DR, Blomhoff R (2006). Content of redox-active compounds (ie, antioxidants) in foods consumed in the United States. Am J Clin Nutr.

[45] Gutteridge JM, Halliwell B (2000). Free radicals and antioxidants in the year 2000. A historical look to the future. Ann N Y Acad Sci.

[46] Moskaug JO, Carlsen H, Myhrstad MC, Blomhoff R (2005). Polyphenols and glutathione synthesis regulation. Am J Clin Nutr.

[47] Lamming DW, Wood JG, Sinclair DA (2004). Small molecules that regulate lifespan: evidence for xenohormesis. Mol Microbiol.

[48] Mellen PB, Walsh TF, Herrington DM (2008). Whole grain intake and cardiovascular disease: a meta-analysis. Nutr Metab Cardiovasc Dis.

[49] Fang L, Li W, Zhang W, Wang Y, Fu S (2015). Association between whole grain intake and stroke risk: evidence from a meta-analysis. Int J Clin Exp Med.

[50] Tang G, Wang D, Long J, Yang F, Si L (2015). Meta-analysis of the association between whole grain intake and coronary heart disease risk. Am J Cardiol.

[51] Aune D, Norat T, Romundstad P, Vatten LJ (2013). Whole grain and refined grain consumption and the risk of type 2 diabetes: a systematic review and dose-response meta-analysis of cohort studies. Eur J Epidemiol.

[52] Chanson-Rolle A, Meynier A, Aubin F, Lappi J, Poutanen K, Vinoy S, Braesco V (2015). Systematic Review and Meta-Analysis of Human Studies to Support a Quantitative Recommendation for Whole Grain Intake in Relation to Type 2 Diabetes. PLoS One.

[53] de Munter JS, Hu FB, Spiegelman D, Franz M, van Dam RM (2007). Whole grain, bran, and germ intake and risk of type 2 diabetes: a prospective cohort study and systematic review. PLoS Med.

[54] Haas P, Machado MJ, Anton AA, Silva AS, de Francisco A (2009). Effectiveness of whole grain consumption in the prevention of colorectal cancer: meta-analysis of cohort studies. Int J Food Sci Nutr.

[55] Schisterman EF, Cole SR, Platt RW (2009). Overadjustment bias and unnecessary adjustment in epidemiologic studies. Epidemiology.

[56] Kyro C, Skeie G, Dragsted LO, Christensen J, Overvad K, Hallmans G, Johansson I, Lund E, Slimani N, Johnsen NF, Halkjaer J, Tjonneland A, Olsen A (2012). Intake of whole grain in Scandinavia: intake, sources and compliance with new national recommendations. Scand J Public Health.

[57] Ross AB, Kristensen M, Seal CJ, Jacques P, McKeown NM (2015). Recommendations for reporting whole-grain intake in observational and intervention studies. Am J Clin Nutr.

[58] U.S. Food and Drug Administration (FDA) Health Claim Notification for Whole Grain Foods.

[59] Stroup DF, Berlin JA, Morton SC, Olkin I, Williamson GD, Rennie D, Moher D, Becker BJ, Sipe TA, Thacker SB (2000). Meta-analysis of observational studies in epidemiology: a proposal for reporting. Meta-analysis Of Observational Studies in Epidemiology (MOOSE) group. JAMA.

[60] DerSimonian R, Laird N (1986). Meta-analysis in clinical trials. Control Clin Trials.

[61] Il'yasova D, Hertz-Picciotto I, Peters U, Berlin JA, Poole C (2005). Choice of exposure scores for categorical regression in meta-analysis: a case study of a common problem. Cancer Causes Control.

[62] Greenland S, Longnecker MP (1992). Methods for trend estimation from summarized dose-response data, with applications to meta-analysis. Am J Epidemiol.

[63] Orsini N, Li R, Wolk A, Khudyakov P, Spiegelman D (2012). Meta-analysis for linear and nonlinear dose-response relations: examples, an evaluation of approximations, and software. Am J Epidemiol.

[64] The Whole Grains Council What is an Ounce Equivalent?.

[65] Begg CB, Mazumdar M (1994). Operating characteristics of a rank correlation test for publication bias. Biometrics.

[66] Egger M, Davey Smith G, Schneider M, Minder C (1997). Bias in meta-analysis detected by a simple, graphical test. BMJ.

[67] Higgins JP, Thompson SG (2002). Quantifying heterogeneity in a meta-analysis. Stat Med.

