# Supplementary material for: Association between whole grain intake and all-cause mortality: a meta-analysis of cohort studies
Source: Oncotarget. 2016 Aug 22;7(38):61996–2005. doi: 10.18632/oncotarget.11491 (PMC5308706; doi:10.18632/oncotarget.11491)
Supplement: Supplementary file 1 [file oncotarget-07-61996-s001.pdf]

Association between whole grain intake and all-cause mortality: a meta-analysis of cohort studies

Supplementary Materials

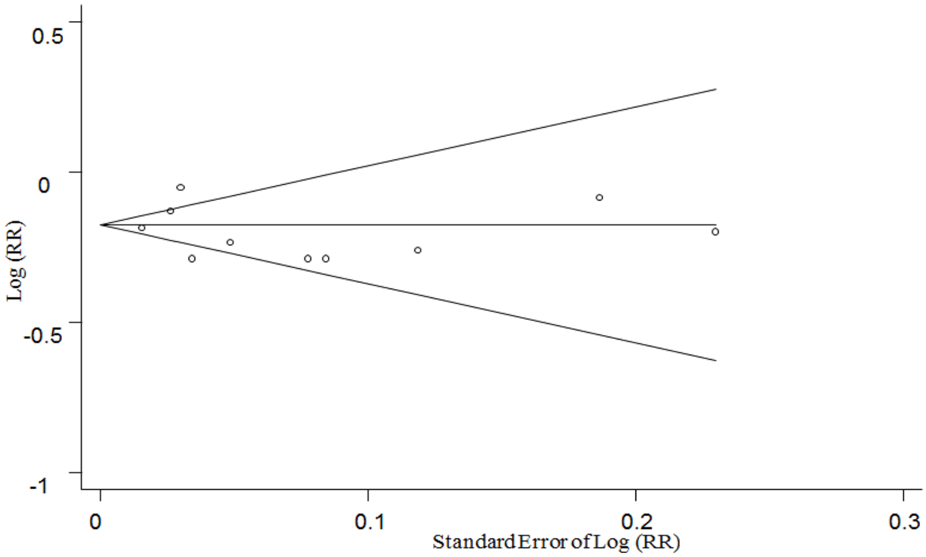

Supplementary Figure S1: Funnel plot of the association between whole grain intake (highest category vs. lowest) and all-cause mortality, 2001–2016.

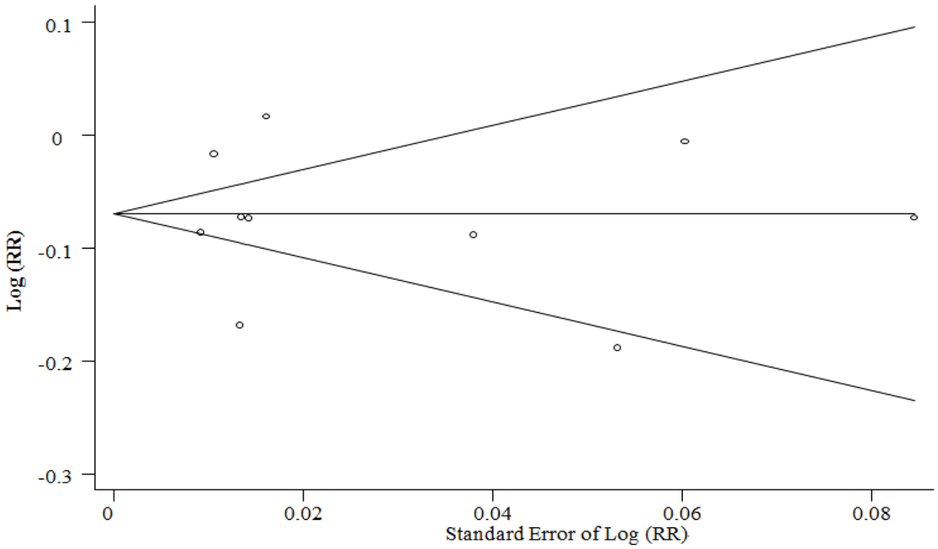

Supplementary Figure S2: Funnel plot of the association between whole grain intake (each 1 serving/d increase) and all-cause mortality, 2001–2016.

Supplementary Table S1: Characteristics of cohort studies included in the meta-analysis, 2001–2016. See Supplementary_Table_S1.

REFERENCES

1. Jacobs DR, Jr., Meyer HE, Solvoll K. Reduced mortality among whole grain bread eaters in men and women in the Norwegian County Study. Eur J Clin Nutr. 2001; 55:137–143.
2. Steffen LM, Jacobs DR, Jr., Stevens J, Shahar E, Carithers T, Folsom AR. Associations of whole-grain, refined-grain, and fruit and vegetable consumption with risks of all-cause mortality and incident coronary artery disease and ischemic stroke: the Atherosclerosis Risk in Communities (ARIC) Study. Am J Clin Nutr. 2003; 78:383–390.
3. Sahyoun NR, Jacques PF, Zhang XL, Juan W, McKeown NM. Whole-grain intake is inversely associated with the metabolic syndrome and mortality in older adults. Am J Clin Nutr. 2006; 83:124–131.
4. Jacobs DR, Jr., Andersen LF and Blomhoff R. Whole-grain consumption is associated with a reduced risk of noncardiovascular, noncancer death attributed to inflammatory diseases in the Iowa Women's Health Study. Am J Clin Nutr. 2007; 85:1606–1614.
5. van den Brandt PA. The impact of a Mediterranean diet and healthy lifestyle on premature mortality in men and women. Am J Clin Nutr. 2011; 94:913–920.
6. Buil-Cosiales P, Zazpe I, Toledo E, Corella D, Salas-Salvado J, Diez-Espino J, Ros E, Fernandez-Creuet Navajas J, Santos-Lozano JM, Aros F, Fiol M, Castaner O, Serra-Majem L, et al. Fiber intake and all-cause mortality in the Prevencion con Dieta Mediterranea (PREDIMED) study. Am J Clin Nutr. 2014; 100:1498–1507.
7. Boggs DA, Ban Y, Palmer JR and Rosenberg L. Higher diet quality is inversely associated with mortality in African-American women. J Nutr. 2015; 145:547–554.
8. Huang T, Xu M, Lee A, Cho S and Qi L. Consumption of whole grains and cereal fiber and total and cause-specific mortality: prospective analysis of 367,442 individuals. BMC Med. 2015; 13:59.
9. Johnsen NF, Frederiksen K, Christensen J, Skeie G, Lund E, Landberg R, Johansson I, Nilsson LM, Halkjaer J, Olsen A, Overvad K and Tjonneland A. Whole-grain products and whole-grain types are associated with lower all-cause and cause-specific mortality in the Scandinavian HELGA cohort. Br J Nutr. 2015; 114:608–623.
10. Wu H, Flint AJ, Qi Q, van Dam RM, Sampson LA, Rimm EB, Holmes MD, Willett WC, Hu FB and Sun Q. Association between dietary whole grain intake and risk of mortality: two large prospective studies in US men and women. JAMA Intern Med. 2015; 175:373–384.
