# Supplementary material for: Association between whole grain intake and all-cause mortality: a meta-analysis of cohort studies
Source: Oncotarget. 2016 Aug 22;7(38):61996–2005. doi: 10.18632/oncotarget.11491 (PMC5308706; doi:10.18632/oncotarget.11491)
Supplement: Supplementary file 2 [file oncotarget-07-61996-s002.docx]

Supplemental Table 1 Characteristics of cohort studies included in the meta-analysis, 2001-2016

| Study | Country | Cohort | Follow-up period | Gender | Age | Size of cohort | All-cause deaths | Exposure | Exposure details | Relative risks (95%  confidence intervals) | Exposure assessment | Adjustments |
| --- | --- | --- | --- | --- | --- | --- | --- | --- | --- | --- | --- | --- |
| Wu et al. 2015 (32) | USA | NHS | 26y | Female | 38-63 years | 74,341 | 15,106 | Whole grain | 4.2 g/d  9.7  14.7  21.1  33.0 | 1.00  0.98 (0.93-1.03)  1.00 (0.95-1.05)  0.94 (0.89-0.99)  0.88 (0.84-0.93) | 126-item FFQ | Age, ethnicity, BMI, smoking status, alcohol intake, physical activity, multivitamin use, aspirin use, a family history of heart disease, a family history of cancer, a family history of diabetes, and history of hypertension, high cholesterol, diabetes at baseline, total energy, modified alternative healthy eating index, menopausal status, and postmenopausal hormone use. |
| Wu et al. 2015 (32) | USA | HPFS | 24y | Male | 32-87 years | 43,744 | 11,814 | Whole grain | 5.9 g/d  14.4  22.1  31.3  47.8 | 1.00  1.00 (0.94-1.05)  0.97 (0.91-1.02)  1.01 (0.95-1.07)  0.95 (0.89-1.00) | 133-item FFQ | Age, ethnicity, BMI, smoking status, alcohol intake, physical activity, multivitamin use, aspirin use, a family history of heart disease, a family history of cancer, a family history of diabetes, and history of hypertension, high cholesterol, diabetes at baseline, total energy, modified alternative healthy eating index |
| Johnsen et al. 2015 (31) | Denmark Norway Sweden | HELGA Cohort | 16y | Both | 30-64 years | 119,518 | 7,839 | Total whole-grain types | Female  20 g/d  33  49  74  Male  21 g/d  37  54  80 | 1.00  0.80 (0.73-0.87)  0.74 (0.67-0.81)  0.74 (0.67-0.81)  1.00  0.82 (0.75-0.90)  0.72 (0.66-0.78)  0.75 (0.68-0.82) | 88-item FFQ (Norway),  98-item FFQ (Sweden)  173-item FFQ (Denmark) | Age, follow-up time, education ,smoking intensity , alcohol intake , BMI, and total energy intake |
| Huang et al. 2015 (30) | USA | NIH-AARP | 14y | Both | 50-71 years | 367,442 | 46,067 | Whole grain products | 0.13 oz/d  0.30  0.47  0.69  1.20 | 1.00  0.93 (0.90–0.95)  0.89 (0.87–0.92)  0.85 (0.82–0.87)  0.83 (0.81–0.86) | 124-item FFQ | Age, gender, the number of cigarettes smoked per day, time of smoking cessation, race or ethnicity group, alcohol intake, education level, marital status, health status, obesity, physical activity, consumption of red meat, total fruit and total vegetables, total energy intake, hormone usage |
| Boggs et al.2015 (29) | USA | BWHS | 16y | Female | 30-69 years | 37,001 | 1,678 | Whole grain products | 0.01 serv/d  0.11  0.32  0.62  1.44 | 1.00  0.96 (0.83-1.12)  0.82 (0.70-0.97)  0.85 (0.73-0.99)  0.75 (0.64-0.89) | 68-item FFQ | Age, each DASH component, total energy intake, education, marital status, vigorous exercise, television watching, smoking, and alcohol intake |
| Buil-Cosiales et al. 2014 (28) | Spain | PREDIMED study | 5.9y | Both | 55-75 years | 7,216 | 425 | Whole grain products | 0 g/d  5  19  84 | 1.00  0.78 (0.52-1.17)  0.67 (0.43-1.04)  0.92 (0.64-1.33) | 137-item FFQ | Age, sex, smoking status, diabetes, BMI, baseline systolic and diastolic arterial blood pressures, and intervention group and stratified by recruitment center use of statins, alcohol intake, educational level, physical activity, total energy intake, fruit and vegetable consumption. |
| van den Brandt. 2011 (27) | Netherland | NLCS (Case-cohort) | 10y | Both | 55-69 years | 3,576 (120,852) | 9,691 | Whole grain products | Female  Per 13.5 g/d  Male  Per 10.6 g/d | 1.00 (0.98-1.03)  1.01 (0.99-1.02) | 150-item FFQ | Age, cigarette smoking status, number of cigarettes smoked per day, years of smoking, BMI, nonoccupational physical activity, history of hypertension, highest level of education, and energy intake |
| Jacobs et al. 2007 (26) | USA | IWHS | 17y | Female | 55-69 years | 27,312 | 5,552 | Whole grain products | 1.75 serv/wk  5.5  9  14.75  23.25 | 1.00  0.88 (0.81-0.96)  0.88 (0.81-0.96)  0.80 (0.73-0.87)  0.79 (0.72-0.87) | 127-item FFQ | Age, energy intake, BMI, waist-hip ratio, smoking, education, physical activity, estrogen use, multivitamin supplement use, and Intakes of alcohol, refined grain, coffee, red meat, fish and seafood, and total fruit and vegetables. |
| Sahyoun et al. 2006 (25) | USA | Not reported | 14y | Both | 60–98 years | 535 | 185 | Whole grain products | 0.31 serv/d  0.86  1.49  2.90 | 1.00  1.08 (0.71-1.66)  1.24 (0.83-1.86)  0.82 (0.52-1.28) | 3-day food  record | Age, sex, race, education, marital status, smoking, alcohol intake, exercise, BMI, energy intake, SFA, antihypertensive or lipid-lowering medication |
| Steffen et al. 2003 (24) | USA | ARIC | 11y | Both | 45-64  years | 11,940 | 867 | Whole grain products | 0.1 serv/day  0.5  1.0  1.5  3.0 | 1.00  0.96 (0.79-1.17)  0.80 (0.65-0.99)  0.87 (0.70-1.08)  0.77 (0.61-0.97) | 61-item FFQ | Age, race, sex, and time-dependent energy intake. education, smoking status, pack-years of smoking, physical activity, alcohol intake, and hormone replacement in women, BMI, waist-to-hip ratio, systolic blood pressure, and use of antihypertensive medications |
| Jacobs DR et al. 2001 (23) | Norway | NCS | 14.4y | Both | 35-56 years | 33,848 | 2,058 | Whole grain bread score | 0.05-0.60 point  0.83-0.83  0.90-1.13  1.35-1.80  2.25-5.40 | 1.00  0.87 (0.75-1.01)  0.80 (0.71-0.92)  0.85 (0.74-0.98)  0.75 (0.65-0.88) | 66-item FFQ | Age, energy intake, sex, smoking, physical activity, use of cod liver oil, multivitamins, SFA intake, systolic blood pressure, serum total cholesterol, BMI |

BMI, body mass index; FFQ, food frequency questionnaire; HPFS, Health Professionals Follow-up Study; NHS, Nurses’ Health Study; HELGA Cohort: Norwegian Women and Cancer Study, Northern Sweden Health and Disease Study, Danish Diet, Cancer and Health Study - part of the EPIC study; NIH-AARP, National Institutes of Health-American Association of Retired Persons; BWHS, Black Women’s Health Study; PREDIMED, Prevencion con Dieta Mediterranea; NLCS, Netherlands Cohort Study; IWHS, Iowa Women’s Health Study; ARIC, Atherosclerosis Risk in Communities Study; NCS, Norwegian County Study;
